# An Ex Vivo Model of Intervertebral Disc Degeneration for Assessing Retention of Injectable Cell‐Based Grafts

**DOI:** 10.1002/jsp2.70144

**Published:** 2025-11-26

**Authors:** Raphael Schmid, Janhavi Apte, Elias Schulze, Andrej Sirek, Günther Schäfer, Jessica Schäper, Francesco Santini, Simona Negoias, Andrea Barbero, Ivan Martin, Karoliina Pelttari, Stefan Schären, Olga Krupkova, Arne Mehrkens

**Affiliations:** ^1^ Spine Surgery University Hospital Basel Basel Switzerland; ^2^ Department of Biomedicine University Hospital Basel and University of Basel Basel Switzerland; ^3^ Institut für Pathologie University Hospital Basel Basel Switzerland; ^4^ Basel Muscle MRI (BAMM), Radiological Physics University Hospital Basel Basel Switzerland; ^5^ Department of Otolaryngology University Hospital Basel Basel Switzerland

**Keywords:** cell therapy, Chondroitinase ABC, ex vivo model, intervertebral disc degeneration, nasal chondrospheres (NCS), pro‐inflammatory cytokines, spheroids

## Abstract

**Introduction:**

Cell therapies for painful intervertebral disc (IVD) degeneration (IDD) have not yet achieved widespread clinical adoption. Understanding therapeutic cell effects in native IVD remains challenging due to the complex IVD environment and limitations of current models. We present a physiologically relevant ex vivo model of IVD degeneration, which we employ to evaluate the retention of therapeutic cells in the IVD.

**Methods:**

Bovine IVDs were cultured ex vivo for 14 days. IVD degeneration was induced under physiological loading by chondroitinase ABC (ChABC), or ChABC with pro‐inflammatory cytokines (Infl) aiming to mimic IDD. The nucleus pulposus (NP) tissue integrity was characterized by T2 MRI and modified Thompson grading and compared with human IVDs of different ages. The onset of IDD in the bovine model was assessed by IL‐8, MMP13, and COX‐2 expression. Spheroids derived from mCherry‐transduced human nasal chondrocytes (NCS) were injected into the NP. NCS retention within the IDD model was assessed by NCS ability to survive (c‐caspase 3), localize (mCherry), and produce chondrogenic proteins (SOX‐9, aggrecan).

**Results:**

ChABC injection reduced water and proteoglycan content in the NP, resembling human age‐related IVD degeneration. ChABC + Infl treatment led to a more pronounced loss of tissue integrity and upregulation of IL‐8, MMP13, and COX‐2, typically characterizing the transition to IDD. Upon injection into the IDD model, NCS localized in the NP, some remained viable, and maintained their chondrogenic features, demonstrating successful retention within the 7‐day time frame.

**Conclusion:**

We developed an ex vivo IVD model with a controlled and physiologically relevant environment and used it for assessing the retention of cell‐based therapies for NP repair. The model recapitulated the progression of IVD degeneration, establishing its value as a preclinical research tool and reducing the reliance on animal studies during the early translational phase.

## Graphical abstract

An ex vivo model of intervertebral disc (IVD) degeneration was generated using chondroitinase ABC (ChABC) and pro‐inflammatory cytokines (Infl) and used for assessing retention of injectable cell‐based grafts, namely nasal chondrocyte spheroids (NCS).

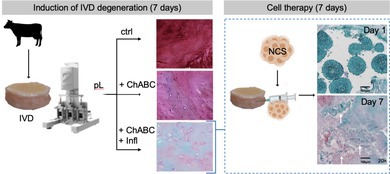

## 1 Introduction

Intervertebral disc disease (IDD) is a progressive disorder that significantly contributes to disability and reduced quality of life in industrialized countries [1]. IDD is characterized by molecular and structural alterations of the intervertebral disc (IVD), which change the load distribution and reduce spine flexibility [2, 3, 4]. It has been estimated that up to 42% of low back pain (LBP) originates from the IVD [5, 6, 7]. IDD is linked with the breakdown of proteoglycans (PG) and collagens in the nucleus pulposus (NP), leading to decreased hydration and structural weakness. Locally released pro‐inflammatory cytokines (e.g., TNF‐α, IL‐1β, IL‐6) along with upregulated matrix degrading enzymes (MMPs, ADAMTSs) exacerbate extracellular matrix (ECM) degradation, allowing for subsequent nerve ingrowth and sensitization. Upregulation of the cyclooxygenase (COX‐2) pathway participates in pain generation by producing prostaglandins and further promoting inflammation [8, 9]. Mechanical stress contributes to microdamage and accelerates cellular senescence and apoptosis, further reducing the reparative capacity [10, 11, 12, 13, 14].

IDD is commonly treated with analgesics and surgery (e.g., spine fusion), but these options are limited to relieving symptoms, carry risks of adverse effects and a high likelihood of recurrence [5]. Due to the limited therapeutic efficacy, there is a growing need for treatments that aim to regenerate the IVD. A suggested therapeutic approach is based on the use of autologous or allogenic cells that restore IVD function by producing ECM and/or by secreting trophic and anti‐inflammatory factors that promote endogenous tissue repair [13, 15, 16, 17, 18, 19]. However, despite significant research, cell therapies have yet to achieve widespread clinical adoption. While some clinical trials showed a reduction of discogenic pain and improved disc height, reproducible and lasting effects such as pain relief with MRI‐detectable repair, could not be reproducibly demonstrated [20, 21, 22, 23]. An important factor that may impair the clinical benefit of cell‐based therapies is the lack of functional integration of the cellular graft, as direct evidence of long‐term survival of therapeutic cells in large animal models or humans remains limited [20].

Degenerated NPs display a unique composition and a catabolic, pro‐inflammatory, and hypoxic microenvironment, often detrimental for the viability and function of therapeutic cells [2, 19]. However, there is still a lack of established preclinical models that would recapitulate these features in a physiologically relevant manner, making it difficult to assess cell behavior in the native tissue in preclinical settings [2, 7, 19, 24, 25]. We thus aim to (i) develop an ex vivo model that simulates staged IVD degeneration and (ii) use this model to study the retention of previously developed therapeutic chondrospheres [26, 27] within NP tissue. Given its high biological relevance, the model could function as an ex vivo optimization tool for cell therapies, potentially lowering the failure rates of cell therapies in clinical trials.

## 2 Materials and Methods

### 2.1 Experimental Design

**FIGURE 1:**
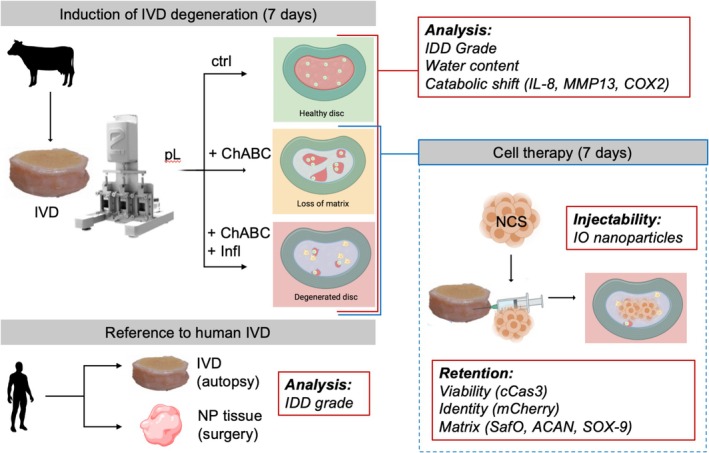
Experimental design. IVDs were isolated from fresh bovine tails and cultured ex vivo. IVD degeneration was induced in a bioreactor under compressive loading (i) by ChABC injection (to induce loss of proteoglycans), and (ii) by ChABC and pro‐inflammatory cytokines (Infl, to induce IDD phenotype). The NP tissue quality was characterized and compared with human IVDs. Human therapeutic cells were then injected as spheroids (NCS) in ChABC‐induced degenerated bovine IVDs and their localization in the NP was confirmed by magnetization with iron oxide (IO) nanoparticles. Cell retention was evaluated by NCS ability to localize in the IVD and express key extracellular matrix markers. ctrl = control, pL = physiological loading, ChABC = Chondroitinase ABC, NCS = spheroids derived from human nasal chondrocytes (NC). cCas3 = cleaved caspase 3, ACAN = aggrecan, SafO = safranin O staining.

The experimental design is outlined in Figure 1. IVDs were isolated from bovine tails and cultured for up to 14 days ex vivo. The ex vivo culture consisted of two phases. In the first phase, IVD degeneration was induced for 7 days under dynamic loading and in the presence of enzymatic digestion, alone (to resemble age‐related IVD degeneration) or with pro‐inflammatory cytokines (to resemble IDD features). In the second phase, therapeutic cells in the form of nasal chondrocyte spheroids (NCS) were injected into the centre of NP cultured under free swelling conditions with native anatomical confinement. The cells were tracked radiologically or by immunofluorescence.

### 2.2 Bovine Intervertebral Disc Organ Culture Model

#### 2.2.1 IVD Degeneration Induction

Fresh bovine tails were obtained from a local slaughterhouse and processed as described before [26]. Whole IVDs (*n* = 3 donors per condition, 3 IVDs per tail) containing only cartilaginous endplates were isolated and cultured in complete IVD culture medium (composition in the Supporting Information) in a sterile container overnight. The next day, IVDs from each donor were randomly divided into three groups and injected with 100 μL of solution containing either PBS (control group), 0.5 U Chondroitinase ABC (ChABC, Sigma Aldrich, 9024‐13‐9), or 0.5 ChABC and pro‐inflammatory cytokines IL‐1β (Sigma Aldrich, SRP3083), TNF‐α (Sigma Aldrich, H8916), and IL‐6 (Sigma Aldrich, SRP3096) (all 100 pg/mL) [28]. ChABC was used to mimic age‐related removal of chondroitin sulfate from proteoglycans (PG) [29, 30] and low‐grade cytokine cocktail was used to induce catabolic responses typical for painful IDD [13, 31, 32]. The solutions were injected using spinal needles (22G, BD405149). The injection volume was administered slowly under constant pressure over a period of approximately 5 min per IVD. Following the injection, the needle was left in place for an additional 2 min to allow for pressure equilibration and tissue accommodation. To minimize potential leakage and promote re‐engagement of the annulus fibrosus (AF) tissue, the needle was carefully withdrawn using a controlled spiral motion. The IVDs were then placed in bioreactor (Ebers TC‐3F) and cultured in IVD culture medium under physiological dynamic loading (PL, 8 h cyclic loading 0.25 Hz and 0.2 MPa, 16 h rest [33]) at 37°C with 5% CO_2_ and 20% O_2_ for 7 days. Medium was changed every 3 days. On day 7, the degeneration status was analyzed by T2 MRI, histological scoring, immunostaining and ELISA.

#### 2.2.2 Cell Therapy Phase

After the degeneration phase, IVDs were injected with 100 (±10%) nasal chondrospheres (NCS, described below) using a spinal needle (22G, BD405149). Individual NCS were first collected from 96‐well plate and pooled into a single 15 mL Falcon tube. The tubes were centrifuged at 1500 rpm for 4 min, after which the supernatant was carefully removed using a pipette. The NCS were then resuspended in 100 μL of PBS, which served as the injection medium. The injection speed varied depending on the internal pressure within each IVD. Prior to injection, the needle was marked at the depth corresponding to the NP tissue to ensure consistent placement. The midpoint of the AF was then determined to guide needle insertion to the pre‐marked depth. In the target depth the NCS were slowly released into the IVD, the syringe was detached, and a metal rod was inserted into the needle to ensure no residual NCS remained within the needle. The needle was retained in place for an additional 5 min to allow for material integration and tissue adaptation. Finally, the needle was withdrawn using a spiral motion to minimize extrusion and encourage re‐assembly of AF tissue. Following injection, IVDs were incubated at 37°C with 5% CO_2_ and 20% O_2_ for up to 7 days. Injected IVDs were harvested at several timepoints to localize the cells upon injection (Day 1, 20 injected NCS, by iron oxide magnetization) and track cell retention (Day 1 and 7, 100 injected NCS, by immunofluorescence and histology).

### 2.3 Preparation of Therapeutic Cells Before Injection

#### 2.3.1 Generation of Nasal Chondrospheres (NCS)

Spheroids generated from human nasal chondrocytes (NC), referred to as nasal chondrospheres (NCS), were used as candidate therapeutic cells. Following local ethical committee approval (EKNZ 2015‐305) and informed consent, human nasal septal cartilage was collected from donors undergoing a rhinoplasty (total *n* = 3 donors, used individually). NC were isolated by digestion in collagenase type II (0.15%, 22 h), resuspended in culture medium, and expanded as described before [26, 34]. NC in passage 1 were transduced with mCherry, to distinguish them from NP cells in IVD tissue. Lentiviral transduction was performed according to an established protocol, affecting neither proliferation nor the differentiation capacity of chondrocytes [35, 36]. Briefly, NC were seeded in 6‐well plates as 2.5 × 10^6^ cells/well and transduced with mCherry‐lentivirus at MOI 1 in the presence of 8 μg/mL polybrene, which provides ≥ 95% transduction efficiency (TEf). TEf was monitored by flow cytometry (Aria III, BD) 3 days post‐transduction. Transduced cells were transferred into T150 flasks and cultured until confluence in standard conditions to ultimately achieve 10^7^ cells per T150. Following transduction, and expansion, NCS were formed from 12′500 NC/spheroid in good manufacturing practice (GMP)‐compliant spheroid microplates (Corning, 4520) for 3 days in chondrogenic medium (composition in Supporting Information) together with 500 ng/mL interleukin 1 receptor antagonist (IL‐1Ra, Peprotech, 200‐01RA) in hypoxia (2% O_2_). This previously established process allows for reproducible generation of NCS with diameters between 300 and 600 μm [26, 27]. For subsequent injection into the IVD, individual NCS were carefully collected from the microplate and pooled into a single 15 mL tube to facilitate loading into the syringe. A tube typically contained one injection dose of 20 NCS/100 μL PBS (for IO labeling on day 1) and 100 NCS/100 μL PBS (for the immuno/histological analysis on day 1 and 7), the latter to reach a total of 1.25 × 10^6^ theoretically injected cells.

#### 2.3.2 Magnetization of NCS With Iron Oxide (Fe_2_O_3_)

Magnetization of NCS with Iron oxide nanoparticles (IO) was performed as described before with slight modifications [37, 38]. Specifically, 50 ng/mL or 150 ng/mL IO magnetic nanoparticles solution (747408‐10 mL, Sigma Aldrich) was mixed with NC expansion medium and added 3 days before NCS formation. After formation, NCS were washed 2 times with PBS. NCS viability and gene expression (described below) were tested and compared to untreated NCS to ensure no particle interference in cellular processes. The 20 IO NCS were then injected into bovine IVDs that underwent IDD induction and cultured in sterile containers at 37°C and 20% O_2_ in a separate experiment. One day after injection, IVDs were imaged under T2 MRI (described below).

#### 2.3.3 NCS Viability Before Injection

Viability of NCS was tested using CellTiter‐Glo Luminescent Cell Viability Assay (Promega, G7570). NCS (*n* = 10 per donor) were transferred to an opaque walled 96‐well plate and 100 μL CellTiter‐Glo 3D Reagent was added per well containing 100 μl of medium plus NCS. Control wells contained only medium plus reagent. The plates were vortexed for 2 min and incubated for a further 10 min. Luminescence was recorded using a 96‐well plate reader (Synergy H1) and viability was estimated based on the signal in treated vs. untreated cells.

#### 2.3.4 NCS Gene Expression Before Injection

One plate (96) NCS was dissociated with 1.5% collagenase at 37°C for 1 h with agitation to dissolve matrix and extract cells. RNA was extracted using Zymo Quick‐RNA Miniprep kit (Cat.no. R1055) according to the manufacturer's instructions. One microgram of RNA was transcribed to cDNA using Invitrogen SuperScript Double‐Stranded cDNA Synthesis Kit. RT‐PCR was carried out using TaqMan primers (Hs00234387_m1 for cCaspase‐3, Hs00264051_m1 for Col2A1, Hs00233992_m1 for MMP13 and Hs02758991_g1 for GAPDH as reference gene) on 7500 Fast Real‐Time PCR System from Thermo Scientific. Data is shown as relative fold change to control (2^−ΔΔCT^).

### 2.4 Human Intervertebral Discs

#### 2.4.1 Autopsy IVDs

Intact lumbar IVDs were collected during regular autopsies from donors (*n* = 4, 1 IVD per donor, Table 1) without a known history of IDD and donating their bodies to research, with informed consent and ethical approval in place (EKNZ‐2023‐02150). Autopsies were conducted within 24 h of death. A spine lumbar motion segment was isolated, aseptically transported to the lab, and cleared with a scalpel. IVDs with cartilage endplates were extracted using a large bone saw (DePuy, Synthes) with ACRION^tm^ saw blades, washed with PBS, and directly fixed for histological scoring.

**TABLE 1:** Bovine and human IVDs used in this study.

	Age	Sex	Level	Degeneration grade	Tissue integrity loss
Bovine IVDs					
PL	5–6 m	M	Cd	1 (T)	Healthy
ChABC	5–6 m	M	Cd	2–3 (T)	Mild/Mod
ChABC + Infl	5–6 m	M	Cd	3–4 (T)	Mod/Severe
Autopsy IVDs					
HuA1	27	M	L2/3	2–3 (T)	Mild/Mod
HuA2	64	F	L4/5	3 (T)	Mod
HuA3	80	M	L3/4	4 (T)	Severe
HuA4	88	M	L2/3	4 (T)	Severe
Surgery IVDs					
HuS1	38	M	L5/S1	2–3 (T), 3 (P)	Mild/Mod
HuS2	42	F	L4/5	4 (T), 4 (P)	Severe

*Note:* In bovine IVDs, IDD was induced by Chondroitinase ABC (ChABC) and pro‐inflammatory cytokines (Infl) under physiological loading (PL). Human NP tissue was obtained from donors undergoing autopsies (HuA1‐4) and surgeries (HuS1‐2).

Abbreviations: Cd, caudal; L, lumbar; Mod, moderate; *P*, Pfirrmann; T, Thompson.

#### 2.4.2 Surgical Nucleus Pulposus (NP) Tissue

Lumbar NP tissue was obtained from patients undergoing surgeries for IDD (*n* = 2, Table 1), with ethical approval (EKNZ‐2015‐305) and informed consent [26, 27]. Tissues were aseptically transported to the lab, washed with PBS, and directly fixed for histological scoring. Modified Thompson grades of surgical NP tissues were compared with Pfirrmann grades obtained with pre‐surgery MRI [39], to verify histological scoring accuracy.

### 2.5 IVD Analytical Techniques

#### 2.5.1 MRI T2 Quantification

##### 2.5.1.1 MRI

MRI T2 quantification measures the T2 relaxation times of the tissue within the IVD, providing information on IVD structural integrity and hydration levels, crucial for maintaining IVD health. MRI scans were performed with a 3 T scanner (MAGNETOM Prisma, Siemens Healthineers, Erlangen, Germany). Immediately after 7 days of culture, intervertebral discs were inspected and put in a standard 50 mL plastic container in PBS for the post‐experimental scan. The plastic container with discs was fixed horizontally in a 24‐channel head coil and placed in the MRI scanner. The central axis of the disc was aligned with the B0 magnetic field of the MRI scanner, minimizing the orientational dependence of the relaxation times caused by dipolar interactions of fiber‐bound water molecules. The MRI protocol comprised a 2D multi spin‐echo sequence for T2 mapping. Acquisition parameters were as follows: Orientation = axial, number of slices = 9, slice thickness = 2 mm, field‐of‐view (FOV) = 90 × 180 mm^2^, in‐plane resolution = 0.7 × 0.7 mm^2^, TR = 5000 ms, TE = 8.6, 17.2, 25.8, 34.4, 43, 51.6, 60.2, 68.8, 77.4, 86, 94.6, and 103.2 ms, acquisition time = 9 m 36 s.

##### 2.5.1.2 IVD Region Definition and Image Analysis

Calculation of the parametric maps was performed in MATLAB R2019a (The MathWorks Inc., Natick, MA). Quantitative T2 values were calculated by pixel‐wise fitting of signal intensities *S* to the equation *S*(TE) = *S*_0_ e^−TE/T2^. Subsequently, the central slice of the stack of 9 slices through the middle of the intervertebral disc was used for further evaluation. A region of interest (ROI) outlining the nucleus pulposus was manually drawn in MATLAB. Quantitative parameter values of T2 are reported as mean ± standard deviation within the ROI. The coding sequence can be found in Supporting Information.

#### 2.5.2 Histology and Immunohistochemistry

##### 2.5.2.1 Modified Thompson Grading

Semiquantitative histological scoring of NP tissue was based on Thompson grading detailed by Rutges et al. [40] and carried out on Safranin O stained histological images. Bovine and human IVDs were fixed in 4% paraformaldehyde at room temperature for 5 days, processed for 28 h using Tissue Processor (TPC 15 DUO, Medite, Germany) and embedded in paraffin. Samples were cut in 6 μm‐thick sections using sliding microtome Microm HM 430E (Thermo Scientific, Germany), and mounted on poly‐L‐lysine coated glass slides. After dehydration, safranin‐O/fast green (SafO/FG; SafO: 84120, Sigma; FG: F‐7252, Sigma) stain with hematoxylin (J.T. Baker, MFCD00078111) nuclear counterstaining was performed to visualize the PG within the sections. Widefield microscopy (Nikon Ti2, Japan; acquisition software: Nikon NIS; Camera: Nikon DS‐Ri2; Objective: ×20 or 40; NA: 0.95) was applied for imaging. For calculation of IVD degeneration grade, three parameters (cellularity, matrix quality, staining intensity) were assessed by three independent scorers and each category received a score between 0 and 2, 0 representing no degenerative characteristics, 1 mild degeneration and 2 severe characteristics of degeneration, as published in^41^. The resulting values were then summarized to calculate the final NP score between 0 (healthy) and 6 (degenerated) and allocated to respective degeneration grades (Table S1). The accuracy of our histological scoring was confirmed on human surgical NP samples by comparing Thompson grade with Pfirrmann grade obtained by pre‐surgery MRI.

##### 2.5.2.2 Immunostaining

Sections were subjected to enzymatic epitope retrieval and blocked with 1% bovine serum albumin (BSA) (A9647, Sigma) supplemented with triton X‐100 (1:1000, 93 418, Sigma), followed by application of primary antibodies, namely anti‐cleaved caspase 3 (1:300, polyclonal, 9661, Cell Signaling; immunoreactive in bovine and human cells), mCherry (1:1500, Rb pAB, AB167453, Abcam), anti‐Aggrecan [6‐B‐4] (1:200, ab3778, Abcam; immunoreactive in human cells), SOX9 (1:100, Rb pAB, NBP2‐24659, Novus Biologicals; immunoreactive in human and bovine cells), MMP13 (1:100, 18 165‐1‐AP, Proteintech; immunoreactive in human cells), COX2 (1:100, MA5‐14568, Thermofisher; immunoreactive in human and bovine cells). Matching secondary antibodies Alexa Fluor 647‐ or 546‐, conjugated (1:500, polyclonal, A21245, Invitrogen and 103‐605‐155, Jackson) were used, with DAPI as a nuclear counterstain. Widefield fluorescence microscopy (Nikon Ti2, Japan; acquisition software: Nikon NIS; Camera: Photometrics Prime 95B; Objective: ×20 or 40; NA: 0.95) was applied for imaging. Images were analyzed in QuPath Bankhead et al. [41]. Regions of interest (ROIs) were manually delineated around the Nucleus Pulposus tissue, avoiding folds, debris, and edge artifacts, depicted as a red circle in the images. For the cell therapy phase, ROI was drawn around mCherry positive cells found in Nucleus Pulposus Tissue. Cell detection was performed on the DAPI channel using QuPath's watershed‐based detector with parameters optimized on representative slides and then kept constant for all images (background radius 8 μm, Gaussian sigma 1.5 μm, nucleus minimum/maximum area 25–400 μm^2^, and cell expansion 3 μm to approximate cytoplasm). For each detected cell, per‐cell mean fluorescence intensity was measured in the target channel(s). During the cell‐retention experiments, Cy5 served as the positivity readout for each cell. Positivity thresholds were defined from negative controls (unstained/secondary‐only regions) and from the lower tail of the intensity distribution within each batch and then fixed across the dataset. Results are reported as the percentage of positive cells per ROI.

#### 2.5.3 Enzyme‐Linked Immunosorbent Assay (ELISA)

Culture medium was collected at the end of IDD phase experiments, representing the last medium change. The amount of IL‐8 in the cell culture medium was quantified, as IL‐8 is known to be released in IVD tissues experiencing a catabolic shift [42, 43]. The Human IL‐8 ELISA Set (555244, BD Biosciences) with the ELISA reagent set B (550534, BD Biosciences) was used to detect IL‐8 according to the manufacturer's instructions [27]. This ELISA kit is highly robust, compatible with our samples, offers an appropriate detection range, and demonstrates cross‐reactivity with bovine IL‐8.

### 2.6 Statistical Analysis

Statistical analysis was performed using GraphPad Prism 9. Data are shown as mean ± SD of samples generated from different donors and biological replicates. One‐way Analysis of variance (One‐way ANOVA) with Tukey's post hoc test was applied to comparisons within sample groups. Statistical differences are considered significant when *p* < 0.05 and indicated with an asterisk (*).

## 3 Results

### 3.1 Degeneration Induction in Bovine IVD Under Loading

With the final goal of investigating the therapeutic cell retention within the native IVD microenvironment, we first developed an organ culture model that recapitulates staged IVD degeneration ex vivo. ChABC without or with pro‐inflammatory cytokines was applied to IVDs cultured in a bioreactor under physiological loading (pL). After 7 days, the structural and biochemical properties of IVDs were assessed, to evaluate tissue integrity and catabolic shift.

#### 3.1.1 Nucleus Pulposus Tissue Integrity

**FIGURE 2:**
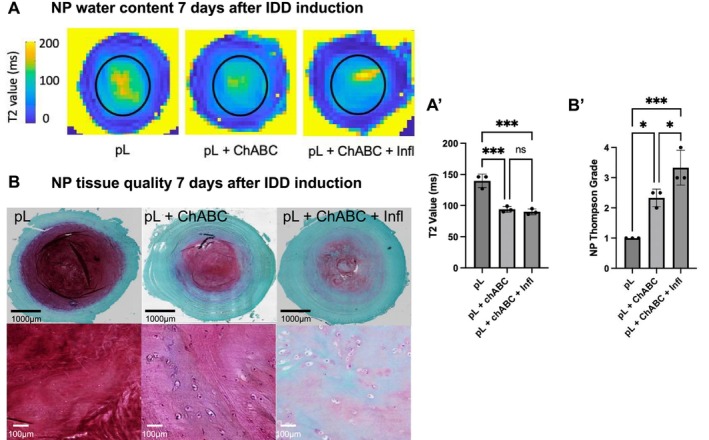
Tissue integrity in bovine ex vivo IVD model. IVDs were injected with 0.5 U Chondroitinase ABC (ChABC) w/wo pro‐inflammatory cytokines (Infl) and subjected to physiological loading (pL) in a bioreactor for 7 days to induce degradative changes. (A) NP water content 7 days after IDD induction: T2 mapping of water content in whole IVD (T2 value, ms) used for (A') quantification of selected ROI (black circles) within NP comparing T2 values (ms) between the groups. Significantly lower T2 values were found in IVDs injected with ChABC and ChABC + Infl compared to pL‐only control (*n* = 3 IVDs per group, mean ± SD, ANOVA). (B) NP tissue quality 7 days after IDD induction: Safranin O and (B′) Modified Thompson grading confirmed increasing tissue/cell disorganization and loss of NP matrix in ChABC and ChABC + Infl groups, compared to pL‐only group (*n* = 3 IVDs per group, mean ± SD). *p < 0.05, **p < 0.01, ***p < 0.001.

Proteoglycans (PG) are the major component of NP ECM, playing a crucial role in maintaining its structure and function by retaining water and osmotic pressure. After the initial IVD degeneration phase, IVDs injected with ChABC and ChABC + Infl showed significantly lower mean T2 relaxation times compared to the pL‐only group (pL 139.6 ms; ChABC 94 ms (*p* = 0.0007); ChABC + Infl 90 ms (*p* = 0.0004), Figure 2A,A'), indicating reduced water content in both degeneration groups. PG in the pL‐only group remained dense and evenly distributed, while IVDs injected with ChABC and ChABC + Infl showed irregular PG distribution and reduced staining intensity, indicating diminished water‐binding capacity (Figure 2B). Modified Thompson grading revealed that NP tissue quality in ChABC and ChABC + Infl groups corresponded to grade 2–3 (mild/moderate degeneration) and 3–4 (moderate/severe degeneration) respectively, demonstrating that different degeneration degrees could be achieved in physiologically loaded bovine IVDs in a short time frame (Figure 2B').

**FIGURE 3:**
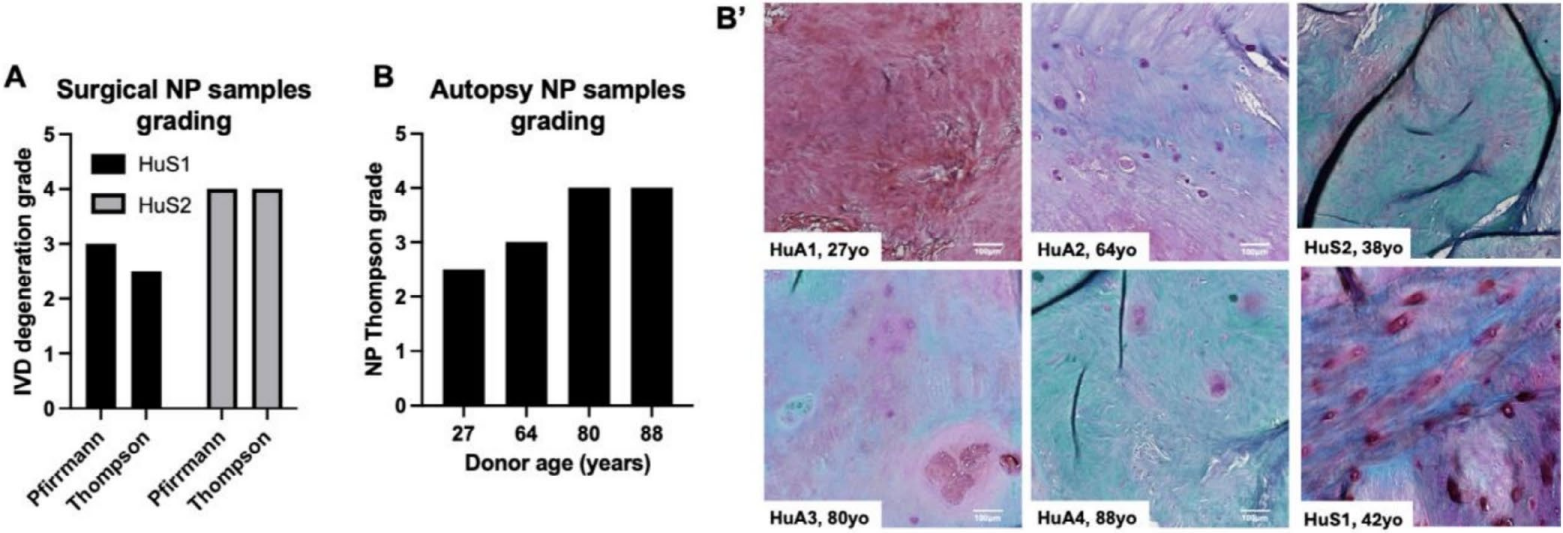
NP tissue integrity in human IVDs. (A) Two NP samples isolated from IDD patients (HuS1‐2) show how the histology‐based Thompson grading corresponds to Pfirrmann grading measured by MRI. (B) In the autopsy NP samples, the IDD grade increased with the age of the donor. (B') Representative NP sections of Safranin O staining, showing the tissue integrity in six human donors of different ages (HuA1‐4: 27, 64, 80, 88 years; HuS1‐2: 38, 42 years).

To compare reduced tissue integrity in our model to that of human IVDs, we analyzed samples obtained from autopsies (HuA) of individuals across age groups as well as surgical specimens (HuS) of IDD patients (Table 1). Surgery NP samples were used to confirm how our assigned modified Thompson grading corresponded to Pfirrmann grading measured by MRI (Figure 3A). In the autopsy NP samples, the IDD grade increased with age of the donor, confirming that the tissue integrity in the ChABC− only group could represent IVD degeneration in middle‐aged individuals, while the ChABC + Infl group could mimic tissue characteristics of elderly IVDs (Figure 3B,B'). Our model thus matched the staged loss of NP tissue integrity found in human IVDs.

#### 3.1.2 Catabolic Shift

**FIGURE 4:**
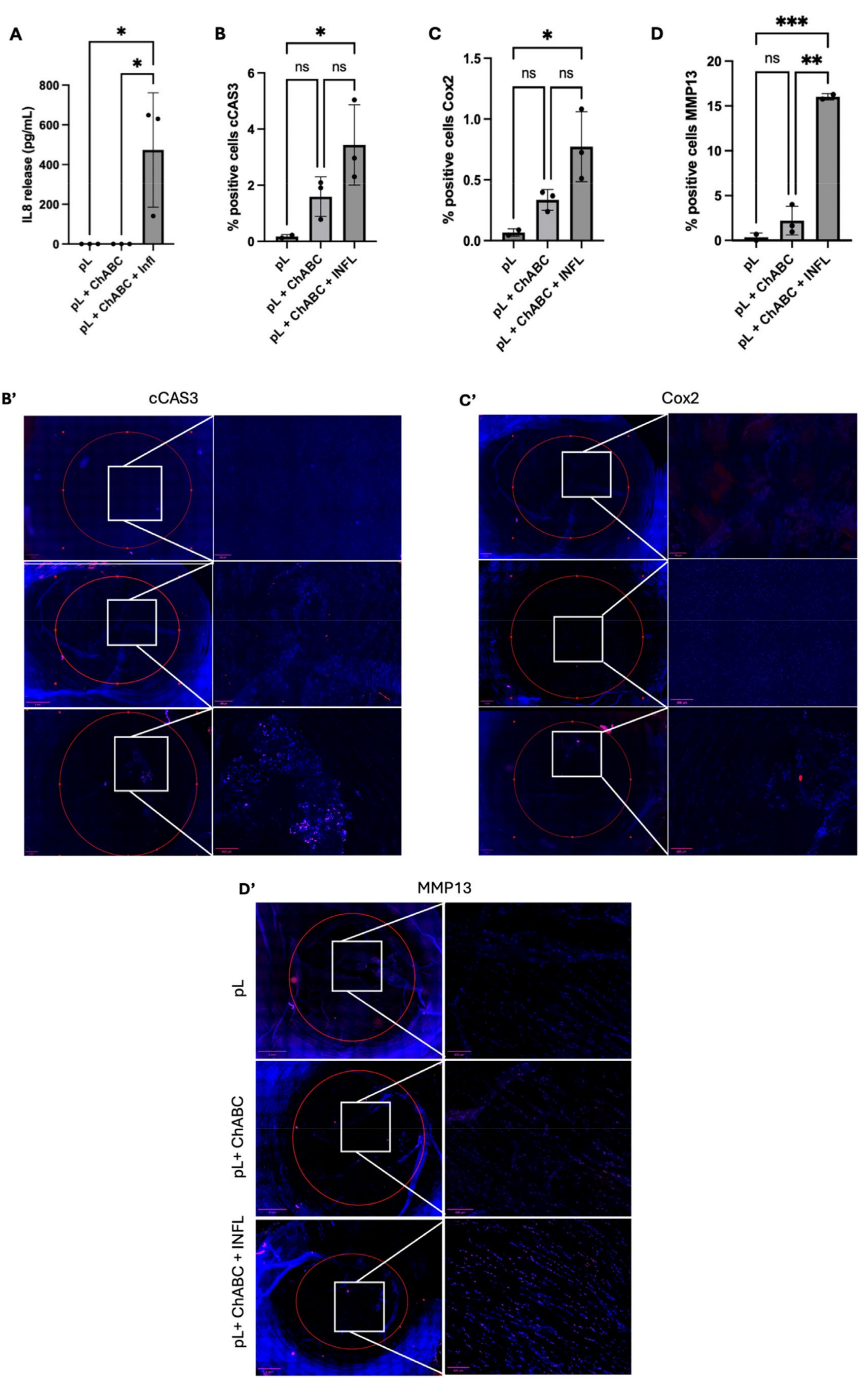
Catabolic shift takes place in IDD‐mimicking conditions. Bovine IVDs treated either with ChABC or ChABC and pro‐inflammatory cytokines (Infl) were cultured under physiological loading (pL), with a pL‐only control group. After 7 days, the ChABC + Infl group upregulated (A) the release of IL‐8 (ELISA), and the expressions of (B) cleavage of caspase 3 (cCAS3), (C) COX‐2, and (D) MMP13. (B′, C′, D′) Representative NP sections of immunofluorescence staining for cCAS3, COX‐2, and MMP13. The experiments were repeated in *n* = 3 donors (mean ± SD, *p* < 0.05, One‐way ANOVA). Scale bar: 400 μm. *p < 0.05, **p < 0.01, ***p < 0.001.

While all individuals may experience IVD degeneration with loss of tissue integrity, not everyone develops discogenic pain. The transition from age‐related to painful IDD occurs when structural damage in NP is accompanied by a catabolic shift that promotes the production of pro‐inflammatory mediators sufficient to trigger pain signaling. This is typically mirrored by increased expression of specific matrix‐degrading enzymes (MMP13), pro‐inflammatory cytokines (IL‐8) and/or pain mediators (COX‐2) [8, 44]. After 7 days of IDD induction ex vivo, bovine IVDs injected with ChABC + Infl showed significantly higher catabolic shift markers such as the release of IL‐8 (*p* = 0.0021, Figure 4A), and the expression of MMP13 (*p* = 0.0013, Figure 4D,D'), with trends toward elevated COX‐2 (Figure 4C,C'), compared to IVDs injected with ChABC only. Cleaved caspase 3 (cCAS3) that marks apoptotic cell death also tended to increase in the ChABC + Infl group, suggesting reduced repair capacity due to the diminishing density of resident cells (Figure 4B,B'). A catabolic shift developed in the ChABC + Infl group, indicating that the onset of features related to IDD requires the presence of inflammation.

In summary, we have established an ex vivo IVD model that can be tuned to replicate the loss of NP tissue integrity typical for age‐related degeneration or to induce the biochemical features associated with IDD.

### 3.2 The Relevance of the Bovine IDD Model for Cell Therapy Testing

**FIGURE 5:**
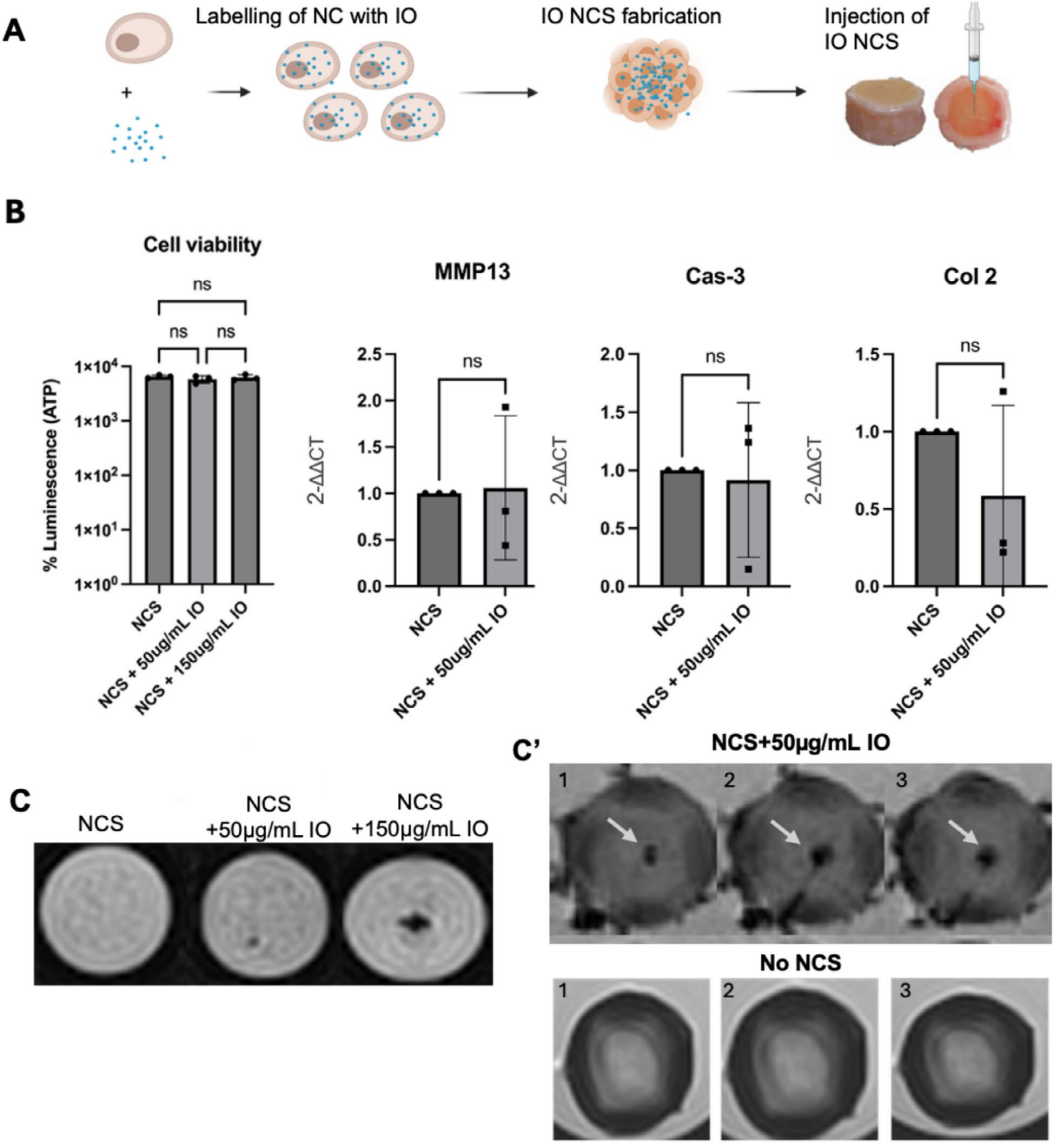
Magnetization of human NCS with Iron oxide nanoparticles (IO). (A) Experimental set‐up for NCS magnetization with 50–150 μg/mL IO. (B) Effects of magnetization on cell viability after 3 days of NCS formation. Gene expression (2^−ddCt^) of COL2A1, MMP13 and Caspase 3 in magnetized NCS vs. non‐magnetized control (NCS). *n* = 3 (mean ± SD, ANOVA, *p*‐value = *n*.s.). (C) MRI: Signal of a single NCS cultured with either 50 or 150 μg/mL of IO in vitro. (C') MRI: The first echoes of three consecutive IVD slices, injected with 20 NCS cultured with 50 μg/mL of IO as well as controls without NCS. Arrows indicate the localization of magnetized NCS within the NP.

Next, we aimed to demonstrate the use of the developed IDD model in testing the retention of therapeutic cells. NCS injectability and localization in the IVD upon injection was evaluated by Iron oxide (IO) nanoparticles, applied in two concentrations on NC before spheroid generation (Figure 5A). NCS magnetization at 50 or 150 μg/mL IO had no influence on cell viability nor the expression of genes relevant for chondrocyte stress and homeostasis, namely COL2A1, MMP13 and caspase 3 (Cas3) (Figure 5B). MRI analysis confirmed that the signal of a single NCS cultured with increasing concentrations of IO is detectable and dose‐dependent (Figure 5C). One day after injecting 20 NCS cultured with the lower IO dose (50 μg/mL), bIVD slices were visualized by MRI. Twenty‐four hours after injection into bovine IVD, NCS localized clearly in the centre of the NP, compared to controls with no NCS injected (Figure 5C'). While this method cannot be used to quantify the number of injected cells, magnetization can help to evaluate differences in formulation injectability and cell localization, allowing us to optimize therapeutic injection strategies (e.g., needle types, injection pumps).

**FIGURE 6:**
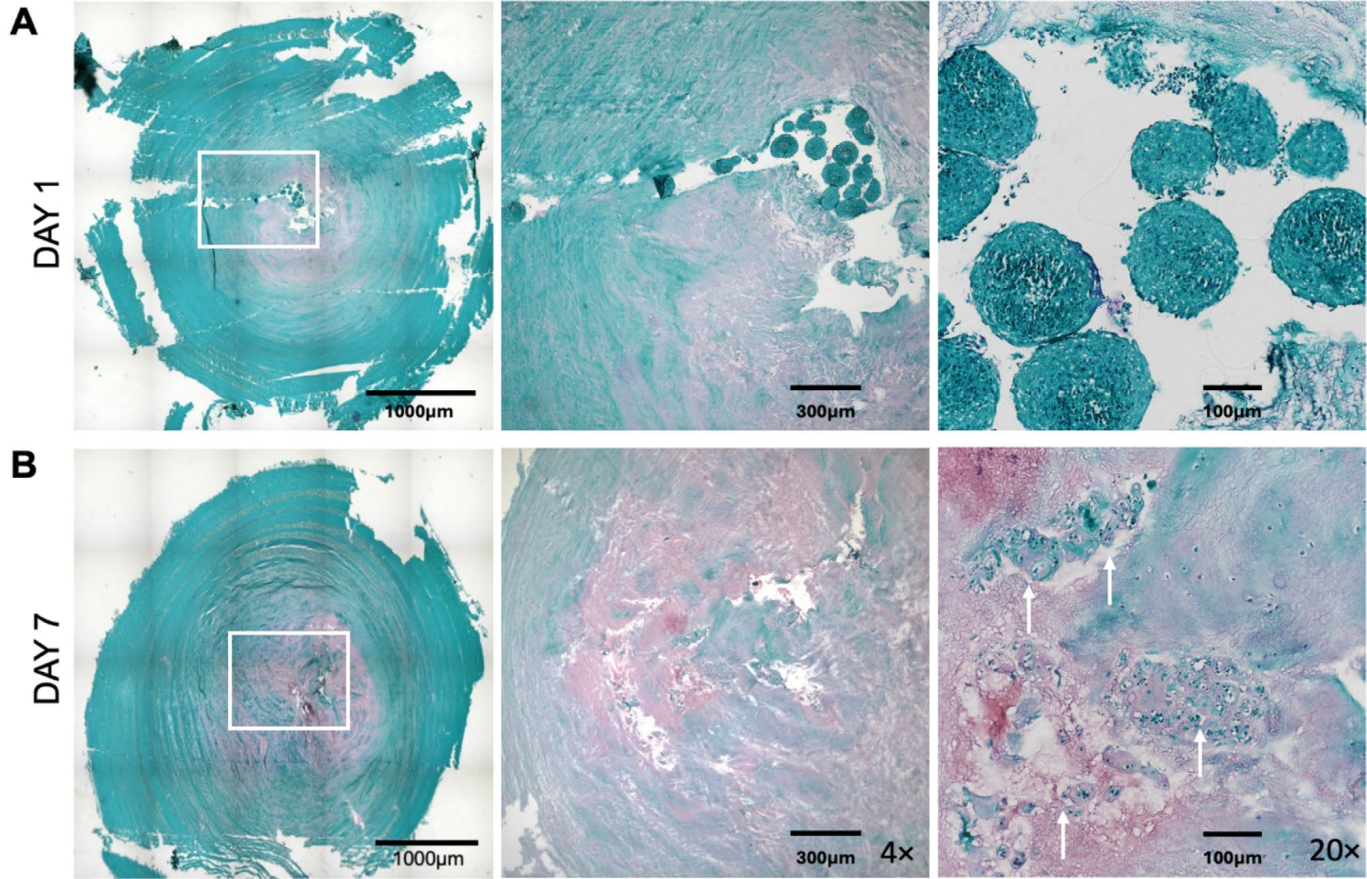
Visualization of human NCS retention in the bovine IDD model. (A) On day 1, injected NCS localized in the NP and maintained its typical round morphology. (B) After 7 days, NCS lost their round shape and appeared to produce matrix (*n* = 3 animals per group).

**FIGURE 7:**
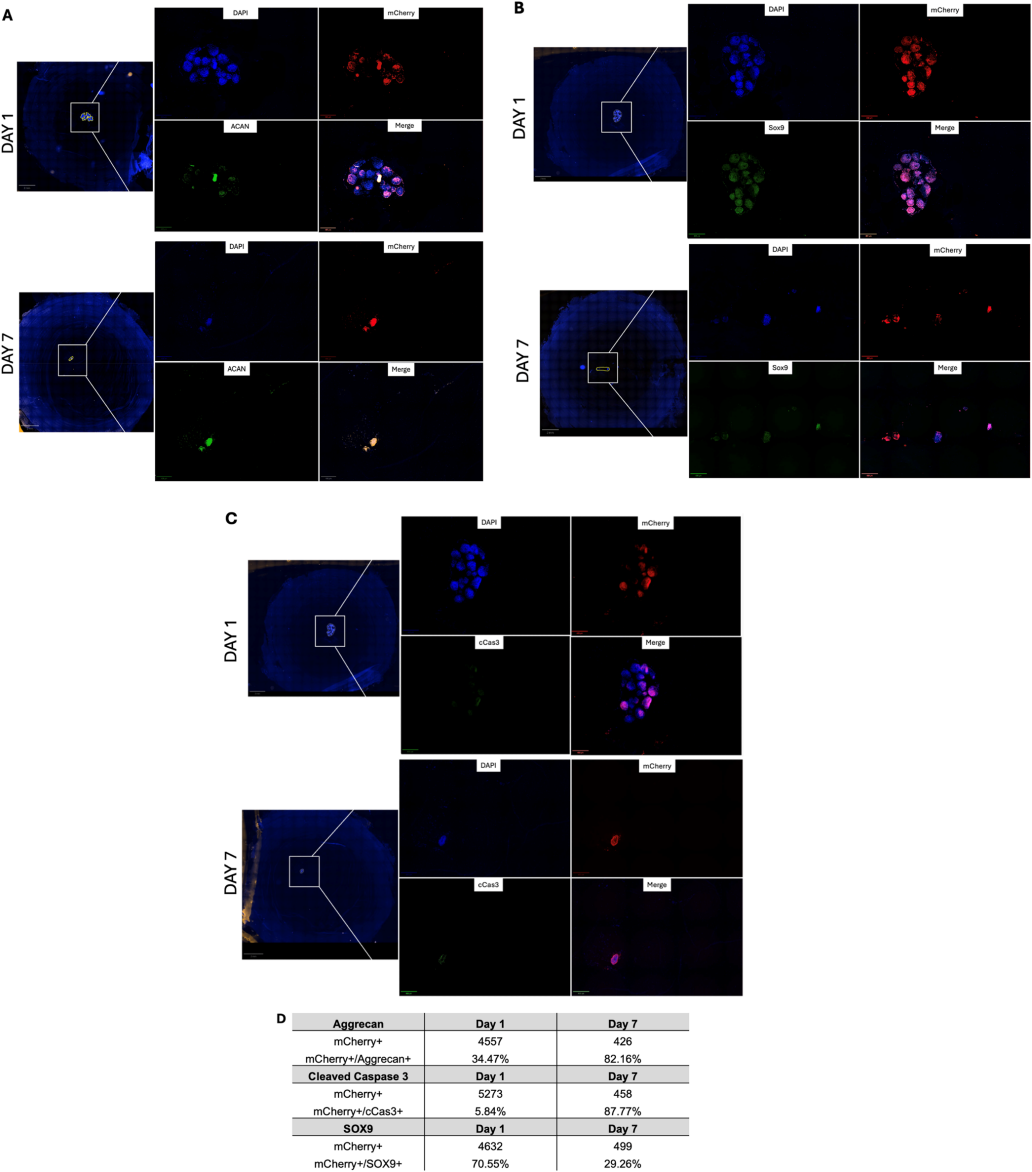
Immunostaining of human NCS retained in bovine IDD model. An expression of (A) aggrecan, (B) SOX‐9, and (C) cleaved caspase 3 (cCas3) in mCherry‐transduced NCS at day 1 and 7 post‐injection (*n* = 3 animals per group). (D) Semi‐quantification of target‐positive cells (%), expressed relative to the total number of cells (DAPI+/mCherry+) within each ROI in a representative histological section. Scale bar: 400 μm.

Ex vivo bovine IVDs treated with ChABC + Infl, where IDD could be recapitulated, were used for NCS testing. For the purposes of our study, we defined the features of cell retention as (i) cell survival, (ii) infiltration into the surrounding tissue, and (iii) preservation of chondrogenic phenotype. Safranin O staining confirmed that injected NCS localized in the NP and maintained their typical round morphology. After 7 days, NCS lost their compact shape and appeared to produce ECM (Figure 6). Immunofluorescence confirmed that mCherry‐expressing NCS localized in the NP. Seven days after their injection into the IVD, a subset of cells appeared to survive (cCas3‐negative, Figure 7C), remain positive for aggrecan (ACAN, Figure 7A), and continue to express SOX‐9 (Figure 7B), although the total number of NCS cells detected in a single histological section had decreased by approximately 10‐fold from day 1 to day 7. Among the remaining cells, over 10% of NCS cells appeared to be viable (Figure 7D), supporting their partial retention in the NP. In summary, our ex vivo bovine IVD model is well suited for proof‐of‐concept testing and optimization of cell therapy studies that require a controlled physiologically relevant IVD microenvironment.

## 4 Discussion

We developed an ex vivo IVD model that successfully replicated key features of IVD degeneration and IDD, by utilizing physiologically relevant stimuli combining mechanical loading, ECM degradation and low‐grade inflammation. Additionally, we provided proof of concept that our IDD model is suitable for testing the retention of injectable cell‐based therapies.

Bovine IVD organ cultures remain widely used for studying IVD degeneration and new therapeutic interventions, including cell therapies [2, 45, 46]. Bovine IVDs have appropriate size, and their widespread availability makes them a practical alternative to fresh human cadaveric samples, which are often limited by ethical and logistical constraints [47, 48, 49, 50]. IDD‐like features are commonly induced either mechanically or chemically, with or without pro‐inflammatory stimuli [26, 28, 50, 51, 52, 53, 54, 55, 56, 57]. The selection of a specific model depends on the intended research purpose and experimental goals, as highlighted in the comparative overview provided in the Supplement (Table S2). Several models utilized degenerative or hyper‐physiological mechanical loading to induce acute damage, making them suitable for studying overload responses but less representative of early‐stage IDD. While prior studies using bovine IVD bioreactors demonstrated that mechanical stress and/or pro‐inflammatory cytokines upregulate inflammation markers, promote ECM degradation, and/or reduce NP cell viability [45, 46, 56, 58, 59, 60], they often entail certain limitations related to their scope or biological relevance for cell therapy testing, prompting the development of our novel setup. Our physiological loading model mimics daily activity in combination with a cocktail of proinflammatory cytokines at physiological concentrations, capturing the gradual inflammatory and catabolic changes characteristic of sustained degeneration, thus providing a biologically relevant context for assessing therapeutic cell retention.

To promote matrix degradation in bovine IVDs, we utilized the degradative effects of a mild lyase enzyme (ChABC). ChABC cleaves chondroitin sulfate residues from proteoglycans, primarily affecting NP GAG content, with delayed effects on collagen content. This process effectively induces the age‐related matrix changes observed in human IVDs in a short time frame [28, 54, 57, 61, 62]. While our ChABC‐only group exhibited ECM disorganization and reduced PG content, its NP cell phenotype remained unchanged. Consequently, ChABC‐only‐treated IVDs provide a model for investigating PG‐related effects such as decreased fixed charge density, altered osmotic pressure, and diminished biomechanical function, which are characteristic of human IVD aging [57].

To better replicate the low‐grade inflammation characteristics of human IDD in our bovine model, we used a combination of three key NP pro‐inflammatory cytokines at biologically relevant concentrations, rather than relying on a single extreme pro‐inflammatory insult [46, 50, 58]. In addition to IL‐1β and TNF‐α, we included IL‐6, which, although not commonly used in this context, plays a biologically relevant role in IDD [63, 64]. This combined approach successfully induced IDD features, characterized by significant increases in IL‐8 and MMP13 in bovine NP tissue [14]. NP cells in the ChABC + Infl group were thus exposed to conditions that impair their function and promote the release of inflammation‐associated molecules in a biologically relevant manner [13, 65].

Tissue retention is essential for the success of cell therapies that aim at supporting the recipient by sustained generation of new ECM and/or delivery of repair signals. Our bovine IDD model was thus designed to assess whether therapeutic cells can withstand the initial microenvironmental changes in the NP and remain within a defect for an initial post‐surgery period. Histological analysis at day 1 post‐injection revealed intact, round NCS, indicating retention after delivery. By day 7, some NCS were no longer visible as discrete round structures. This morphological change appeared to result from a combination of cell loss and possible NCS infiltration into the surrounding NP, consistent with our previous findings showing that NCS spread and fuse with NP microtissues in an IDD‐mimicking environment in vitro [26]. While we observed sustained expression of chondrogenic markers in the retained NCS, extending the culture period could provide more comprehensive insights into the long‐term role of therapeutic cells in ECM remodeling [66, 67].

We employed three complementary methods to track the injected NCS within NP tissue: IO labeling, genetic modification with mCherry, and conventional histological staining. A comparative overview of advantages and limitations of each of these NCS tracking methods is outlined in the Supplement (Table S3). Conventional histological staining is essential for evaluating tissue morphology and matrix composition, but does not fully distinguish between donor and host cells without additional labeling. Thus, the use of IO and mCherry in conjunction with standard histology provided complementary NCS tracking. IO labeling enabled non‐invasive real‐time localization of NCS in the IVD. This approach provided immediate feedback on the accuracy of NCS delivery on a macroscale, while also preserving the phenotype of the ex vivo culture. However, individual spheroids could not be clearly distinguished by MRI because the magnetic signals clustered together. In contrast, mCherry labeling allowed for specific and stable detection of NCS cells via fluorescence microscopy at end point and permitted multiplexing with other immunofluorescent targets. mCherry was essential for distinguishing NCS from native NP cells and for quantifying immunofluorescence signals specifically originating from NCS.

Several limitations must be considered when interpreting the results of our work: First, subtle differences from human in vivo physiology in cellular responses, ECM composition, and IVD anatomy may restrict the direct applicability of the findings to human conditions. While our IDD model contains sterile inflammation within NP tissue, its inherent lack of interactions with the immune system and surrounding tissues does not allow for fully replicating native human IDD. Second, dynamic loading was applied to accelerate the degenerative effects, while static conditions were used during the cell therapy phase to mimic the reduced mobility typically seen post‐surgery. Although this approach is effective for assessing cell retention in proof‐of‐concept investigations, incorporating loading during the cell therapy phase would be necessary to simulate the irregular loading patterns that may occur in degenerated motion segments. Third, our study did not specifically investigate alternatively regulated cell death pathways (e.g., necroptosis, autophagy) [68, 69], thus their contribution to NCS cell loss after transplantation cannot be excluded. Another limitation is the absence of quantitative analysis of the number of iron oxide‐labeled NCS. While MRI allowed us to localize labeled NCS within the IVD, it did not enable reliable quantification due to the 3D nature of the NCS. In regions where labeled cells clustered, the magnetic signal from overlapping cells became indistinct, limiting the ability to differentiate between a high local cell density and a concentrated iron signal from fewer cells [70, 71]. For these reasons, we focused our analysis on qualitative localization of IO‐labeled NCS. Future work incorporating quantitative magnetization‐based imaging modalities (such as magnetic particle imaging, MPI) may allow more precise cell tracking in these complex settings. Lastly, a small sample size limits the statistical power of our findings. As this study focused on establishing feasibility and proof‐of‐concept, the sample size balanced our exploratory aims with practical constraints. Future studies with larger sample sizes are necessary to achieve robust statistical validation of these results.

In summary, our model recapitulated biological changes seen in IDD patients [14], such as ECM degradation and low‐grade inflammatory/catabolic microenvironment. These features mimicked the conditions therapeutic cells would encounter after their implantation in vivo, collectively influencing cell retention potential in native NP tissue [45, 50]. While our model offers an alternative to existing ex vivo models [46, 56], its translational relevance is reinforced by employing physiological degradative stimuli and a real cell‐based scenario, thereby demonstrating its applicability in preclinical IVD research.

## 5 Conclusions

The interactions between therapeutic cells and the IVD microenvironment remain poorly understood, which contributes to reduced survival and functionality of cell therapy products. The ex vivo bovine IVD model presented here successfully replicated IDD, capturing distinct stages of tissue degeneration and catabolic shift in a short timeframe. By combining matrix‐degrading enzyme and low‐grade inflammation under physiological loading conditions, this model provided a biologically relevant and sustainable platform for evaluating the retention of IVD cell therapies within the IDD microenvironment.

## Author Contributions

**Olga Krupkova:** conceptualization. **Raphael Schmid**, **Janhavi Apte**, **Jessica Schäper**, and **Francesco Santini:** methodology. **Raphael Schmid**, **Janhavi Apte**, **Günther Schäfer:** investigation. **Raphael Schmid**, **Janhavi Apte:** formal analysis. **Elias Schulze**, **Andrej Sirek**, **Simona Negoias**, **Andrea Barbero**, **Ivan Martin**, **Stefan Schären:** resources. **Raphael Schmid:** data curation. **Olga Krupkova**, **Raphael Schmid**, **Janhavi Apte:** writing – original draft. **Olga Krupkova**, **Arne Mehrkens**, **Karoliina Pelttari**, **Stefan Schären**, **Ivan Martin**, **Andrea Barbero:** writing – review and editing. **Raphael Schmid**, **Janhavi Apte:** visualization. **Olga Krupkova**, **Arne Mehrkens**, **Karoliina Pelttari:** supervision. **Olga Krupkova**, **Karoliina Pelttari:** project administration. **Ivan Martin**, **Karoliina Pelttari**, **Olga Krupkova**, **Arne Mehrkens**, **Stefan Schären:** funding acquisition.

## Funding

This work was supported by the European Research Council, 810111‐EpiCrest2, Freiwillige Akademische Gesellschaft, FAG‐05/2024, and SwissLife Jubiläumsstiftung, SwissLife‐05/2024.

## Conflicts of Interest

The authors declare no conflicts of interest.

## Supporting information


**Data S1:** Supporting Information.

## Data Availability

The data that support the findings of this study are available on request from the corresponding author. The data are not publicly available due to privacy or ethical restrictions.
