# Supplementary material for: An Ex Vivo Model of Intervertebral Disc Degeneration for Assessing Retention of Injectable Cell‐Based Grafts
Source: JOR Spine. 2025 Nov 26;8(4):e70144. doi: 10.1002/jsp2.70144 (PMC12648434; doi:10.1002/jsp2.70144)
Supplement: Supplementary file 1 — Data S1: Supporting Information. [file JSP2-8-e70144-s001.docx]

**Supplementary material**

**A. Medium composition**

**NC expansion medium:** DMEM (Gibco, 10938-025) containing 10% fetal bovine serum (FBS; Gibco, 12491-015), supplemented with 1 mM sodium pyruvate (SP; Invitrogen, 11360-039), 10 mM HEPES (Invitrogen, 15630-056), 100 U/mL penicillin, 100 µg/mL streptomycin, 0.29 mg/mL L-glutamine (PSG; Invitrogen, 10378-016), 1 ng/mL transforming growth factor (TGF) β1 (R&D, 240-BO-10) and 5 ng/mL fibroblast growth factor (FGF2; R&D, 233-FB-025).

**Chondrogenic medium:** DMEM LG: 2.2% of 4.5g/l G (Gibco, 10938025) + 97.8% of 0g/l G (Gibco, A1443001), 1 mM SP, 10mM HEPES, PSG, 10 μg/mL, Insulin-Transferrin-Selenium (ITS, Gibco, 51300-044), 5.6 mg/L Linoleic acid (LA, Sigma, L9530-5ML), 0.75 g/l human serum albumin (HSA, CSL Behring 20%, 200g/L), 10 ng/mL TGF-β3 (100-36E, Peprotech), 0.1μM dexamethasone (Sigma, D-2915) and 0.1 mM ascorbic acid-2 phosphate (Sigma, A-8960).

**IVD complete medium:** DMEM (Gibco, 10938-025), supplemented with 1 mM SP (Invitrogen, 11360-039), 10mM HEPES, PSG (Gibco, 10378-016), 10 μg/mL ITS, 5.6 mg/L LA, and 0.75 g/l HSA.

**B. Coding sequence of T2-quantified water content within NP-ROI**

Coding sequence:

[X_1,map]=dicomread('image0001.dcm');

[X_2,map]=dicomread('image0002.dcm');

[X_3,map]=dicomread('image0003.dcm');

[X_4,map]=dicomread('image0004.dcm');

[X_5,map]=dicomread('image0005.dcm');

[X_6,map]=dicomread('image0006.dcm');

[X_7,map]=dicomread('image0007.dcm');

[X_8,map]=dicomread('image0008.dcm');

[X_9,map]=dicomread('image0009.dcm');

[X_10,map]=dicomread('image0010.dcm');

[X_11,map]=dicomread('image0011.dcm');

[X_12,map]=dicomread('image0012.dcm');

BILDER = cat(3,X_2,X_3,X_4,X_5,X_6,X_7,X_8,X_9,X_10,X_11,X_12);

mitte_x = squeeze(mean(BILDER,1));

mitte_y = squeeze(mean(BILDER,2));

pixel_x=68;

pixel_y=48;

pixel=double(squeeze(BILDER(pixel_x,pixel_y,:)));

TEs=[2,3,4,5,6,7,8,9,10,11,12];

TE = TEs*8.6;

modelfun = @(params, TE)(params(1)*exp(-TE/params(2))');

t2map = zeros(size(BILDER,1), size(BILDER,2));

for i = 1:size(BILDER,1)

for j = 1:size(BILDER,2)

if BILDER(i,j,1) < 100

continue

end

params = nlinfit(TE, double(squeeze(BILDER(i,j,:))),@t2_model,[1000, 100]);

t2map(i,j) = params(2);

end

end

function y = t2_model(params, te)

y = params(1)*exp(-te/params(2))';

y(isinf(y)) = -1000;

y(isnan(y)) = -1000;

end

For the ROI:

m = roipoly;

nPixels = sum(m(:))

averageT2 = sum(t2map(m)) / nPixels

**C. Modified Thompson grading of bovine and human IVDs**

***Table S1. Thompson grading of bovine and human IVDs.*** *Histological scoring system was based on Rutges at al. (2013)^1^ and slightly modified for Safranin O-stained NP assessed by three scorers.*

| **Score** | **Grade** | **IVD degeneration** | **Description** |
| --- | --- | --- | --- |
| 0 | 1 | Healthy | Well organized NP matrix, no cell clusters, no signs of degeneration. |
| 1-2 | 2 | Mild | Early signs of degeneration, such as slight changes in cellularity and NP matrix. |
| 3-4 | 3 | Moderate | Visible changes in the NP structure, such as more pronounced disorganization and inhomogeneity. |
| 5-6 | 4 | Severe | Significant disorganization and loss of NP matrix, clustered cellularity, chondroid nests present. |

**D. Supporting tables for the discussion**

***Table S2. Comparative overview of bovine ex vivo IVD models that include mechanical loading w/wo IDD-like conditions.***

| **Study**  **(First Author, Year)** | **Model & Loading Regime** | **Deg. Induction Method** | **Duration** | **Main Readouts** | **Key Findings** | **Advantages & Limitations relative to others** | **Source** |
| --- | --- | --- | --- | --- | --- | --- | --- |
| Walter, 2011 | Static compression in culture (0.5 MPa) | Static compressive load | 7 days | Cell viability, GAG content, histology, gene expression | Static loading led to decreased GAG, cell death, and degenerative morphology | **Advantages:** Simple, reproducible model for static load-induced degeneration. **Limitations:** Static loading less physiologically relevant than dynamic or complex loading. | <https://pubmed.ncbi.nlm.nih.gov/21549847/> |
| Chan, 2013 | Loading bioreactor, combined dynamic axial compression (0.2–0.8 MPa, 0.1 Hz) and axial torsion (±2°, 0.1 Hz) | Combined compression and torsion loading | 14 days | Cell viability, gene expression, GAG, collagen, histology | Combined compression and torsion caused greater cell death, more severe matrix degradation, and increased catabolic gene expression than compression alone | **Advantages:** Simulates more physiologically relevant, complex loading; demonstrates synergistic degeneration  **Limitations:** Still lacks inflammatory/biochemical factors; more technically demanding than axial-only loading | <https://pubmed.ncbi.nlm.nih.gov/24013824/> |
| Gawri, 2014 | Axial loading bioreactor, static and dynamic loading (0.1–0.3 MPa, 0.1 Hz, 4 h/day) | Trypsin injection (10 or 100 µg/disc, NP) | 14 days | GAG, disc height, histology, WB, DNA content | Dynamic loading preserved GAG content and disc height after trypsin-induced degeneration, unloaded discs lost matrix and height | **Advantages:** Long-term, viable culture. Useful for distinguishing effects of static vs. dynamic loading. **Limitations:** Reproduction of inflmmation and catabolic shift unclear. | <https://pubmed.ncbi.nlm.nih.gov/24992586/> |
| Chooi, 2016 | Axial loading bioreactor, static (0.35 MPa) and dynamic (0.35 ± 0.25 MPa, 0.2 Hz) | Physiological loading (no explicit injury) | 2 h per session; dynamic loading 2 h/day for 2 days | Cell activity, gene expression, cytoskeleton | Physiological loading upregulated HSP70 without affecting cell activity or matrix remodeling genes | **Advantages:** Good for studying early cellular stress responses. **Limitations:**Does not model degeneration; limited for studying progressive or severe changes. | <https://pmc.ncbi.nlm.nih.gov/articles/PMC5006975/> |
| Zhou, 2020 | Axial loading bioreactor; physiological (0.02–0.2 MPa, 0.2 Hz) vs. "one strike" (50% strain/sec) | Single high-impact compressive load ("one strike") | 1 or 8 days | Cell viability, histology, GAG/NO release, gene expression | "One strike" caused cell death, ECM breakdown, AF fissures, catabolic gene upregulation, modeling traumatic degeneration | **Advantages:** Mimics acute traumatic injury; rapid degeneration induction. **Limitations:** Less representative of chronic degeneration compared to other loading models. | <https://pmc.ncbi.nlm.nih.gov/articles/PMC7773974/> |
| Li, 2020 | Axial loading bioreactor  (control: 0.02-0.2 MPa; 0.2 Hz; 2 h/day + high glucose medium) | High load: 0.32–0.5 MPa @5 Hz, 2 h/day + TNF‑α (100 ng/IVD) + low glucose | 11 days | Inflammatory cytokines, MMP levels,  cell viability, GAG | High load, low glucose, and TNF-α led to inflammatory response, matrix breakdown, and cell death | **Advantages:** Combined mechanical, inflammatory & nutritional stress **Limitations:** High load and TNF‑α may exceed physiological range | <https://pubmed.ncbi.nlm.nih.gov/32587853/> |
| Secerovic, 2022 | 6-DOF bioreactor, multiaxial loading | High-magnitude, complex mechanical loading | Up to 3 weeks | IVD height, cell viability, mechanical properties | High-magnitude/complex loading induced degenerative changes; validated long-term ex vivo culture | **Advantages:** Closest to physiological loading environment; can simulate complex in vivo forces **Limitations:** High technical complexity, lower throughput, expensive setup | <https://pubmed.ncbi.nlm.nih.gov/35977717/> |
| Vernengo, 2023 | Axial loading bioreactor (0.02–0.2 MPa 0.2 Hz, 2 h/day) | Papain, chABC, collagenase II (enzymatic) | 7 days | GAG, disc height, cell viability, gene expression, histology | Enzymes esp. papain) caused distinct matrix loss, height reduction, voids, and some cell death, but no significant inflammatory response | **Advantages:** Compares effects of different enzymes **Limitations:** Void formation not representative of natural IDD, catabolic shift not reproduced | <https://pubmed.ncbi.nlm.nih.gov/37711456/> |

***Table S3. Tracking methods for nasal chondrospheres in nucleus pulposus tissue.*** *Comparison of iron oxide (IO) labeling, mCherry fluorescent protein labeling, and conventional histological staining for tracking injected NCS in NP tissue. The table summarizes the specificity, sensitivity, advantages, and potential limitations of each method.*

| **Method** | **Specificity for Donor Cells / Sensitivity** | **Advantages** | **Limitations** |
| --- | --- | --- | --- |
| Iron oxide (IO) | Moderate / High | No genetic modification required  High labeling efficiency in vitro  Can be used for **in vivo tracking** | **Signal dilution** with cell division or death  Iron may be phagocytosed by host cells, causing **false positives**  Cannot distinguish live from dead cells |
| mCherry fluorescence | High / High | Highly specific and stable labeling  Enables **clear identification of donor cells**  Signal persists through cell divisions  No risk of label transfer to host cells | Requires genetic modification of donor cells  Fluorescence can fade during tissue processing  Autofluorescence of NP tissue can interfere  May affect cell function in some contexts |
| Histological staining | None / None | Essential for assessing tissue morphology | **Cannot distinguish donor from host** cells without additional labeling |

**References**

(1) Rutges, J. P. H. J.; Duit, R. A.; Kummer, J. A.; Bekkers, J. E. J.; Oner, F. C.; Castelein, R. M.; Dhert, W. J. A.; Creemers, L. B. A validated new histological classification for intervertebral disc degeneration. *Osteoarthritis and Cartilage* **2013**, *21* (12), 2039-2047. DOI: <https://doi.org/10.1016/j.joca.2013.10.001>.
